# Supplementary material for: DNA:RNA Hybrids Are Major Dinoflagellate Minicircle Molecular Types
Source: Int J Mol Sci. 2023 Jun 2;24(11):9651. doi: 10.3390/ijms24119651 (PMC10253752; doi:10.3390/ijms24119651)
Supplement: Supplementary file 1 [file ijms-24-09651-s001.zip › ijms-2432530-supplementary.pdf]

Supplementary materials

DNA:RNA Hybrids Are Major Dinoflagellate Minicircle Molecular Types

Alvin Chun Man Kwok †, Siu Kai Leung † and Joseph Tin Yum Wong *

Division of Life Science, The Hong Kong University of Science and Technology, Clearwater Bay, Kowloon, Hong Kong, China; alvink@ust.hk (A.C.M.K.); leungsiukai82@yahoo.com.hk (S.K.L.)
* Correspondence: botin@ust.hk
† These authors contributed equally to this work.

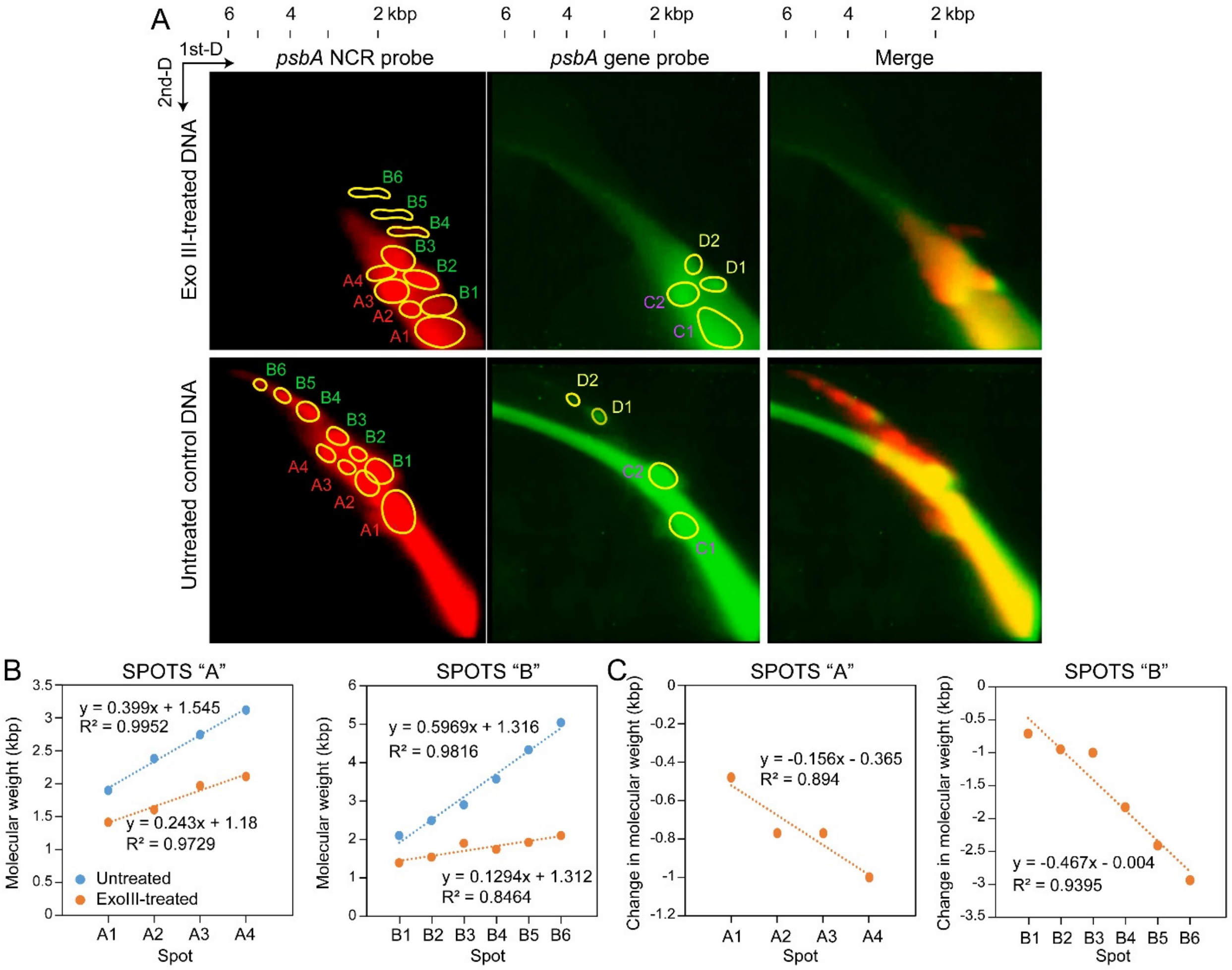

Figure S1. Re-analysis of minicircle intermediates in 2-dimensional gel electrophoresis. (A) Overlay images of the southern blot signals of Exo III-treated whole-DNA preparations from *Heterocapsa triquetra*, which were probed with 1.1-kb *psbA* NCR fragment (red) or *psbA* gene (green). (B). The different slopes (B, C) after ExoIII digestion demonstrated the original spots contained modular molecular types that contained more than one nucleic acids, essentially DNA:RNA hybrids with either DNA or RNA extensions.

Table S1. Primers.

Purpose	PCR annealing temperature (°C)	Name of primer	Sequence (5′ to 3′)
Amplification of the 0.4 kbp-southern hybridization probe to NCR	52	0.4K-F	CTCAGTACTTTTCCCCGGTAA
		R	TAGAATGCAATAAAAATGAACCTAGCTTG
Amplification of the 0.7 kbp-southern hybridization probe to *psbA* gene	51	BA1-COMP	CAGTTTGGGAAGCTCTTGG
		BA5-COMP	GCAAGATCAAGTGGGAAGTTG
Amplification of the 0.6 kbp-southern hybridization probe to NCR	54	F1	TATATGCATTCATAAACCGTCGAAGC
		R2	ACCCCCAAATCTGAGCCCCAG
Single primer PCR	54	F1	TATATGCATTCATAAACCGTCGAAGC
		R	TAGAATGCAATAAAAATGAACCTAGCTTG
Nested PCR following single primer PCR	50	F2	AAACACATGCAATTTGCCTTG
		R3	TGCATTTGGGACTCCACTTTG
		R4	ATACTAGAAATCTATCCATAACAT
		R5	ACAATCAACAAAGCCACTAAC
		R6	AGGTTGGTGTGTGATTTAGCC

Table S3. Repetitive sequences identified on the HtNCR sequences.

Repeats	Sequence (5′ to 3′)	Number of repeat	Repeat length	Location in HtNCR
$(ATT)_3$	ATTATTATT	3	3	134-136, 137-139, 140-142
Repeat 1	AAATCCTGATAAATTTCAC-TTTTCTCAGTACTTTTCCCCGGTAAAAGGGGGGGGTGTCTGCGAT-TTCAAAGTGGAGTCCCAAATGCATCTTCGGAATATATGAGGAG	2	108	174-281, 655-762
Repeat 2	AGATATTTGAAGAT	2	14	305-318, 325-338
Repeat 3	AGATATTTGA	3	10	305-314, 315-324, 325-334
$(TCTA)_3$	TCTATCTATCTA	3	4	476-479, 480-483, 484-487
Repeat 4	AACCTTCGGGT	2	11	966-976, 1017-1027
Repeat 5	GGTTATTATCGGTTA	3	15	1025-1039, 1035-1049, 1045-1059
Repeat 6	GGTTATTATC	3	10	1025-1034, 1035-1044, 1045-1054
